# Oxidation‐Induced Internal Chemical Patterning in Copper–Gold Nanoalloys

**DOI:** 10.1002/smll.75003

**Published:** 2026-08-04

**Authors:** Zhikang Zhou, Hanlei Zhang, Dongxiang Wu, Jianyu Wang, Xiaobo Chen, Chaoran Li, Shan Yan, Jing Li, Adrian Hunt, Iradwikanari Waluyo, Chuanjian Zhong, Guangwen Zhou

**Affiliations:** ^1^ Materials Science and Engineering Program & Department of Mechanical Engineering State University of New York Binghamton New York USA; ^2^ Department of Chemistry State University of New York Binghamton New York USA; ^3^ National Synchrotron Light Source II Brookhaven National Laboratory Upton New York USA

**Keywords:** alloy, chemical physics, copper, density functional theory, diffusion, interface segregation principle, materials science, particle, surface energy, surface oxidation

## Abstract

Chemical segregation governs the structural evolution and properties of nanoalloys and, under vacuum or reducing conditions, typically leads to core–shell structures that minimize surface free energy. Here, we show that surface oxidation fundamentally alters this behavior in Cu–Au nanoalloys by suppressing conventional surface segregation and driving twin‐boundary (TB)‐mediated chemical separation within the particle interior. Combining in situ electron microscopy, ambient‐pressure X‐ray photoelectron spectroscopy, and density functional theory, we reveal that oxidation redirects Au transport from the surface to internal TBs through uphill diffusion from Au‐poor to Au‐rich regions, producing alternating Au‐rich and Cu‐rich lamellae with local Au enrichment of up to ∼50 at.%. This behavior is driven by the negative Cu–Au mixing enthalpy, which favors heteroatomic bonding, while the CuO*_x_* overlayer inhibits Au surface segregation that would otherwise dominate. Consequently, surface oxidation acts as a chemical pump, redirecting atomic flux into the particle interior, where defect‐mediated segregation lowers the free energy by maximizing Cu–Au bonding. These findings demonstrate how surface chemistry, alloy thermodynamics, and lattice defects collectively govern mass transport and internal chemical patterning, providing atomistic insight into the structural evolution and stability of nanoalloys under reactive environments.

## Graphical abstract

Surface oxidation suppresses Au surface segregation and redirects Au transport to twin boundaries, replacing conventional core–shell formation with internal chemical patterning. Driven by Cu–Au mixing thermodynamics, defect‐mediated uphill diffusion creates alternating Au‐rich and Cu‐rich lamellae, revealing oxidation as a chemical pump for bulk compositional patterning.

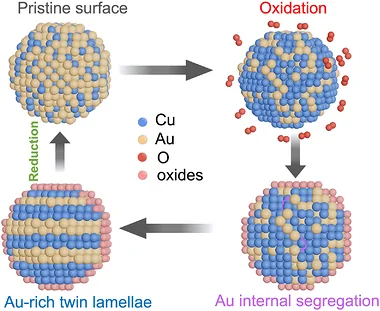

## 1 Introduction

Understanding how elements distribute and reorganize at the nanoscale is fundamental to controlling the stability and functionality of alloy nanoparticles (NPs)  [1, 2, 3]. In multimetallic systems, atomic segregation—the preferential enrichment of one element at particular crystallographic sites—governs catalytic activity, selectivity, and deactivation resistance [4, 5]. For decades, such behavior has been rationalized largely within the “core–shell” paradigm. In archetypal systems such as Cu–Au, Pt–Ni, and Pd–Au, thermodynamics typically favor configurations in which the component with lower surface energy (e.g., Au) segregates to the surface, thereby minimizing the overall interfacial free energy [6, 7, 8, 9, 10, 11]. This surface enrichment is often considered as a prerequisite for optimized catalytic performance, as it creates the geometric and electronic surface ensembles required for active‐site synergy [12, 13, 14].

Under operando catalytic environments, however, nanoalloys rarely retain these equilibrium configurations. Exposure to reactive gases, oxidizing conditions, or thermal cycling can dynamically restructure the lattice and alter compositional distributions [15, 16, 17, 18, 19, 20, 21, 22, 23, 24, 25]. While recent studies have begun to reveal thermally driven interdiffusion, chemical ordering, and phase evolution in multicomponent nanoalloys [26, 27, 28, 29, 30], it remains poorly understood how surface chemistry and bulk crystal defects cooperatively govern internal compositional and structural evolution under reactive atmospheres. This challenge is particularly acute in alloy systems whose constituent elements exhibit strong disparity in chemical reactivity toward gas‐phase species. Such asymmetry often triggers complex, nonequilibrium structural transformations that have remained mechanistically elusive at the atomic scale.

Here, we employ Cu–Au nanoalloys as a model system—characterized by a sharp chemical asymmetry between readily oxidized Cu and oxidation‐resistant Au [31, 32, 33, 34], and relevant to catalytic reactions such as CO oxidation, CO_2_ reduction, and selective hydrogenation [6, 30, 35, 36, 37, 38, 39, 40, 41]—to demonstrate a fundamental departure from the conventional core–shell paradigm. We show that surface oxidation suppresses the thermodynamically expected surface segregation and instead drives twin‐boundary (TB)‐mediated chemical separation within the particle interior. By integrating in situ transmission electron microscopy (TEM), ambient‐pressure X‐ray photoelectron spectroscopy (AP‐XPS), and density functional theory (DFT) calculations, we reveal that oxidation inhibits Au surface enrichment and instead promotes the formation of alternating Au‐rich and Cu‐rich lamellae anchored to TBs—defects ubiquitous in face‐centered cubic (fcc) metals [42, 43, 44, 45, 46, 47].

Remarkably, this internal compositional patterning arises from a counterintuitive “uphill diffusion” of Au atoms, in which Au migrates from Au‐poor regions toward Au‐rich lamellae. This behavior defies conventional expectations and originates from oxidation‐induced modifications to the thermodynamic landscape. Surface oxidation acts as an external driving force that redistributes internal chemical potential, enabling Cu–Au pairwise interactions to drive Au enrichment up to ∼50 at.% within the lamellae domains. Such twinning‐mediated segregation has profound catalytic implications: by dismantling Au‐enriched surface ensembles, it disrupts the synergistic Cu–Au interactions required for catalytic activity, providing a direct atomistic explanation for performance degradation under oxidative conditions. More broadly, these results uncover a previously unrecognized coupling between surface chemistry, alloy thermodynamics, lattice defects, and bulk mass transport in nanoalloys. By bridging surface reactions with internal lattice defect dynamics, this work establishes a predictive framework for understanding how reactive environments reshape nanoalloy architectures—and suggests strategies for either harnessing or mitigating these transformations to control catalyst stability and functionality.

## 2 Results

### 2.1 Oxidation‐Induced Internal Compositional Patterning

Cu–Au alloy NPs are synthesized using a wet chemistry approach (Materials and Methods, Appendix Tables S1–S2, Figure S1). Representative TEM imaging of the as‐synthesized Cu_75_Au_25_ NPs (Figure 1A) reveals nearly spherical particles with diameters ranging from ∼5 to 50 nm. High‐angle annular dark field scanning TEM (HAADF‐STEM) (Figure 1B) identifies a thin native Cu oxide shell surrounding the NPs, the presence of which is further verified by AP‐XPS measurements (discussed later). In the as‐prepared state, low‐magnification HAADF‐STEM imaging (Figure 1C) shows uniform contrast across individual particles, indicative of a homogeneous alloy. This chemically disordered state is further confirmed by atomic‐resolution HAADF‐STEM (Figure 1D), where the uniform intensity of atomic columns and the corresponding Fast Fourier transform (FFT) pattern (inset, Figure 1D) reveal a randomized fcc lattice, consistent with a Cu(Au) solid solution and in agreement with previous reports [48]. Together, these observations establish that the as‐synthesized Cu(Au) NPs adopt a random solid‐solution structure without detectable chemical separation or long‐range ordering.

**FIGURE 1:**
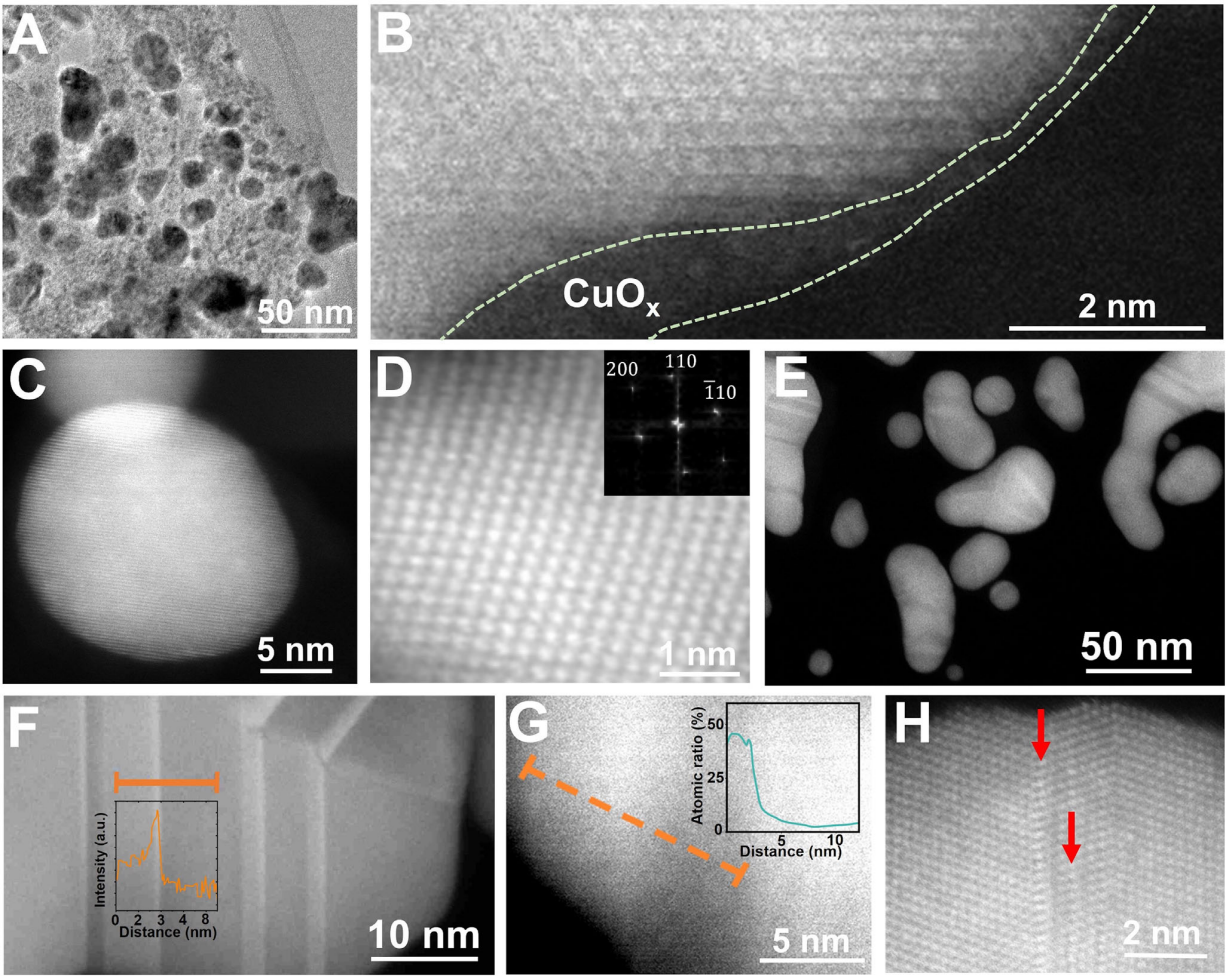
Surface‐oxide‐induced internal chemical patterning in Cu(Au) NPs. (A) Low‐magnification TEM image of the as‐synthesized Cu_75_Au_25_ NPs. (B) HAADF‐STEM image resolving the native CuO*_x_* overlayer on the NP surface. (C) Low‐magnification HAADF‐STEM image demonstrating uniform mass‐contrast across individual NPs (except for locally brighter contrast arising from NP overlap), indicative of a chemically homogeneous initial state. (D) Atomic‐resolution HAADF‐STEM image and the corresponding FFT (inset) confirming a chemically disordered fcc Cu(Au) solid solution. (E) Low‐magnification HAADF‐STEM image of NPs after thermal annealing (∼450°C, ∼1 h), revealing the emergence of pronounced lamellar *Z*‐contrast within the particle interiors. (F) HAADF‐STEM image of a representative NP displaying bright, parallel, and regularly spaced lamellae corresponding to Au‐rich regions. Inset: intensity profile along the orange line across an Au‐rich lamella. (G) HAADF‐STEM image overlaid with an Au concentration profile extracted from an EDS line scan (orange dashed line), providing chemical confirmation of alternating Au‐rich and Cu‐rich lamellae. (H) Atomic‐resolution HAADF‐STEM image resolving Au‐rich atomic columns (higher intensity) preferentially localized along TBs (red arrows). The absence of enhanced intensity at the NP surface (upper region) confirms the suppression of Au surface enrichment, marking a fundamental departure from classic segregation behavior.

Upon thermal treatment, however, the internal architecture of the NPs undergoes a striking reconfiguration. Figure 1E presents an HAADF‐STEM image of Cu_75_Au_25_ NPs after annealing at ∼450°C for ∼1 h under TEM vacuum conditions. In contrast to the homogeneous as‐prepared state, the annealed NPs exhibit pronounced internal contrast variations, manifested as alternating bright and dark lamellae. Given that HAADF‐STEM contrast scales approximately with the square of the atomic number (*Z*‐contrast) [49, 50, 51], these variations directly indicate a phase separation into Au‐rich (bright) and Cu‐rich (dark) domains. High‐magnification imaging of a representative annealed NP (Figure 1F) reveals that these domains organize into parallel, regularly spaced lamellar ensembles extending across the particle interior. Subsequent crystallographic analysis identifies these alternating regions as twin‐related variants. Notably, the Au‐rich lamellae are systematically narrower than the Cu‐rich domains, as quantitatively captured by the line intensity profile in Figure 1F. The relative volumetric fractions of these domains are consistent with the nominal Cu_75_Au_25_ stoichiometry, indicating a redistribution of species within the particle rather than a net loss of Au during annealing.

To quantify the observed compositional modulation, we perform EDS line‐scan analysis across the lamellar features (Figure 1G). The results confirm a striking disparity in local stoichiometry: the Au‐rich lamellae reach a concentration of ∼46 at.% Au at the TB, while the adjacent Au‐depleted regions contain as little as 5 at.% Au. This distribution indicates a robust migration of Au atoms from the bulk toward the TBs, resulting in localized interfacial enrichment. Statistical analysis of ∼200 annealed NPs reveals that 43% of the population develops this periodic lamellar architecture (Figure S2). Crucially, NPs that remain chemically homogeneous were found to be devoid of twinning defects (Figure S3). This strong structural–chemical correlation identifies TBs as the indispensable structural template for facilitating bulk chemical separation.

Atomic‐resolution HAADF‐STEM imaging (Figure 1H) provides further evidence of this unconventional segregation pathway. Specifically, the surface atomic columns exhibit relatively lower intensity than the internal lattice, indicating an absence of Au surface enrichment. Instead, Au atoms are preferentially sequestered at internal TBs, as evidenced by the high‐intensity (*Z*‐contrast) atomic columns localized along these interfacial planes. Collectively, these observations delineate a dramatic structural transition: the as‐prepared Cu–Au NPs, initially characterized by a random solid solution and a native CuO*_x_* passivation layer, undergo a dramatic internal reconfiguration upon vacuum annealing. This process results in the emergence of periodic, alternating Au‐rich and Cu‐rich lamellar twins. This internally confined chemical separation contrasts sharply with classical surface‐driven segregation, which traditionally dictates the formation of core–shell architectures characterized by an Au‐enriched exterior.

### 2.2 Mechanistic Pathway of Twin‐Facilitated Chemical Separation

In situ HRTEM observations reveal that the formation pathway of chemically separated twin lamellae follows a distinct two‐step sequence: structural twinning precedes compositional segregation. Initially, twin lamellae emerge with a uniform Cu(Au) framework; subsequent atomic redistribution across the TB transforms these domains into alternating Cu‐ and Au‐rich regions. A time‐resolved HRTEM sequence (Figure 2A–C and Video S1) captures this evolution in a single‐crystal Cu_75_Au_25_ NP during annealing under TEM vacuum. In its initial state (0 s, Figure 2A), the NP exhibits a homogeneous Cu(Au) solid solution without visible twining or structural defects. Upon heating at 450°C for 163 s (Figure 2B), a TB nucleates via symmetric lattice shearing across the {111} plane. Continued thermal annealing for 860 s (Figure 2C) results in a well‐defined, coherent TB that bisects the crystal into mirror‐symmetrical domains. The corresponding diffractogram (Figure 2D) exhibits two sets of reflections rotated by ∼70.5° (the characteristic angle for fcc twinning), confirming the twin relationship along the [011] zone axis.

**FIGURE 2:**
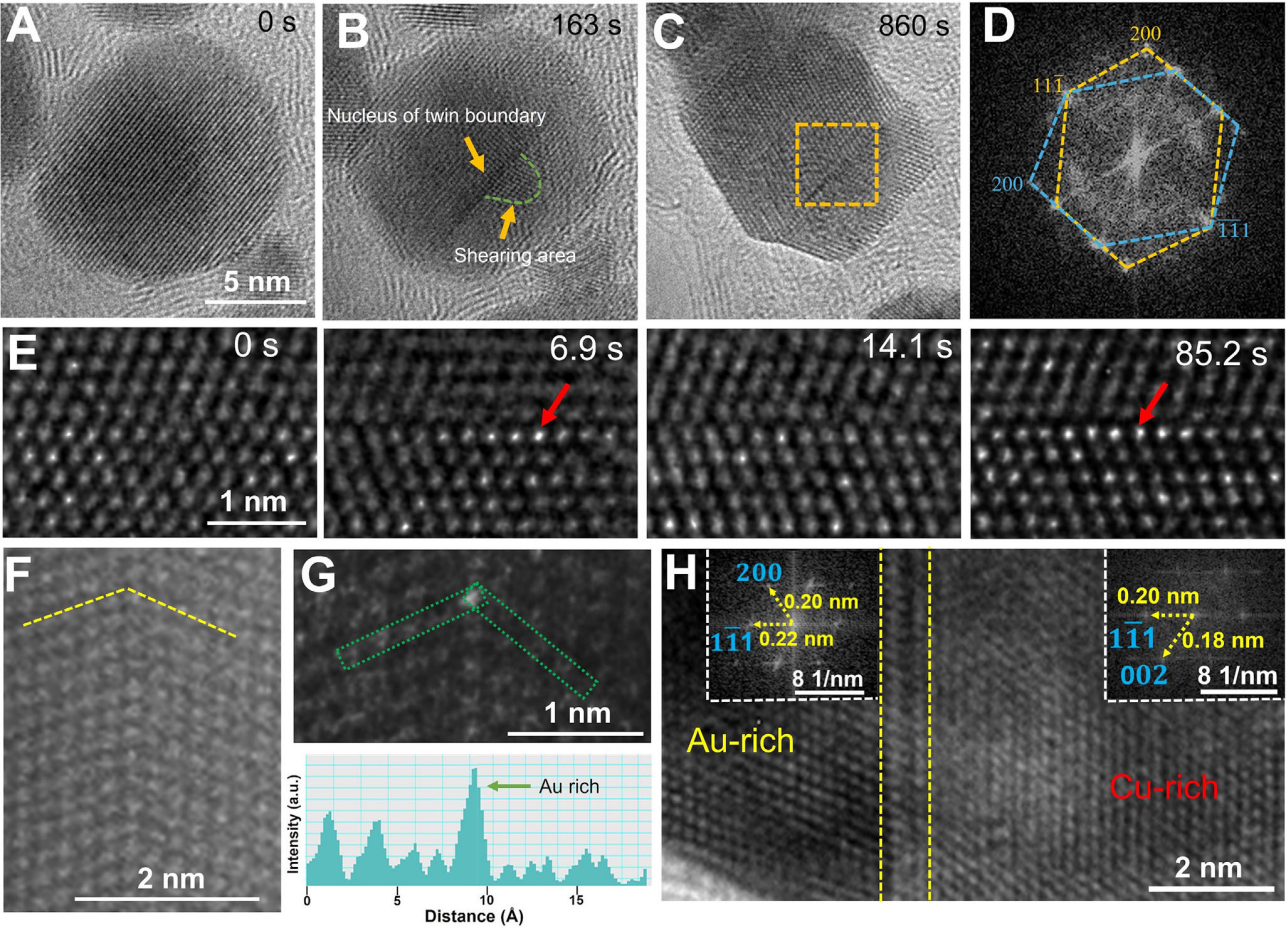
Dynamic evolution and crystallographic signature of twin‐facilitated chemical separation. (A–C) In situ HRTEM time‐sequence (Video S1) capturing the nucleation and stabilization of a coherent TB within a Cu_75_Au_25_ NP during vacuum annealing at 450°C. (D) FFT pattern from the boxed region in (C), indexing the twin relationship across the {111} plane. (E) Time‐resolved HRTEM sequence (Video S2) demonstrating oscillatory flux of Au across the twin interface. The repeated enrichment and depletion cycles at the TB—characterized by transient contrast variations—drive the directional transport of Au from the upper domain into the lower domain. (F) Atomic‐resolution HAADF‐STEM image of a nascent TB prior to chemical separation, exhibiting uniform *Z*‐contrast. (G) HAADF‐STEM image and the corresponding integrated intensity profiles along the dotted green boxes, resolving the emergence of a high‐intensity (Au‐rich) atomic column at the TB, marking the initiation of Au sequestration. (H) HRTEM image following prolonged annealing, showing established Au‐rich and Cu‐rich lamellae separated by TBs (yellow dashed lines). Inset: FFTs resolve a distinct lattice expansion in the Au‐rich domain relative to the Cu‐rich domain, consistent with the larger atomic radius of Au.

The nucleation of the TB is attributed to thermally induced lattice shear, driven by the relaxation of internal strain fields. Such transformations are energetically facilitated by the low stacking‐fault energy characteristic of Cu(Au) alloys [52, 53, 54]. At elevated temperatures, enhanced atomic mobility allows the lattice to accommodate local elastic strains—arising from spatial fluctuations in the Cu/Au distribution [8]; the lattice accommodates these strains through the formation of stacking faults that subsequently evolve into coherent TBs. This structural reorganization reduces the total free energy of the system by relieving bulk elastic strain [55, 56, 57].

Once established, coherent TBs serve as both high‐diffusivity conduits and energetic sinks for Au atoms. This preferential segregation is driven by two synergistic factors: (i) Lowered kinetic barriers: The periodic lattice discontinuities at the TB locally weaken atomic bonding, thereby reducing the activation energy for migration. This allows Au atoms to diffuse along the interface significantly faster than through the bulk lattice. (ii) Strain‐driven thermodynamics: Twinning introduces tensile–compressive strain fields adjacent to the boundary. Because the atomic radius of Au is larger than that of Cu, these strain fields energetically favor the sequestration of Au atoms at the boundary, where local lattice expansion accommodates the atomic size mismatch. These mechanisms make TBs efficient conduits for Au transport and highly favorable sites for Au accumulation. Once sufficient enrichment occurs, the boundary serves as the nucleus from which alternating Au‐rich and Cu‐rich lamellae develop. The continued Au enrichment within the Au‐rich lamella is driven by the thermodynamic preference for Cu–Au pairwise interactions relative to Cu–Cu and Au–Au interactions, promoting the migration of Au from the Cu‐rich domain through the TB toward the Au‐rich domain, as further supported by our DFT modeling described later.

The proposed mechanistic framework is directly corroborated by in situ HRTEM imaging of a Cu_75_Au_25_ NP during vacuum annealing at ∼450°C (Figure 2E and Video S2). High‐resolution tracking of a single TB resolves the real‐time redistribution of solute atoms. At *t* = 0 s, the lattice contrast is relatively uniform across both the TB and the adjacent twin domains, indicating a homogeneous initial composition. After 6.9 s, several atomic columns along the TB exhibit a discernible increase in intensity (Figure 2E, red arrows), signaling the onset of Au sequestration at the interface. Concurrently, the adjacent upper twin domain undergoes a loss of contrast, reflecting localized Au depletion as atoms migrate toward the interfacial sink. By 14.1 s, the boundary contrast attenuates as Au redistributes from the TB into the adjacent lattice of the lower twin domain, which exhibits a corresponding increase in contrast. Simultaneously, the upper domain shows a partial recovery in brightness relative to its state at 6.9 s, indicating a transient replenishment of Au flux from distal lattice sites beyond the immediate field of view.

Following extended thermal treatment (*t* = 85.2 s), the system exhibits a secondary brightening of the TB and a subsequent dimming of the upper domain. This suggests a renewed flux of Au from the Cu‐rich upper domain toward the TB. Meanwhile, the lower twin domain shows a progressive and sustained increase in image contrast relative to its initial state, confirming a net accumulation of Au over time. This observed cycle of enrichment and depletion at the TB—coupled with the monotonic Au accumulation in the lower lamella—demonstrates that the TB functions as a high‐diffusivity “conduit” and an energetic pump. Au atoms are harvested from the Cu‐rich lamella (upper domain), transmitted through the boundary, and incorporated into the growing Au‐rich lamella (lower domain). This dynamic redistribution identifies the TB as the active structural template that directs the chemical separation into alternating Cu‐rich and Au‐rich lamellar ensembles within the NP interior. This “capture‐and‐release” behavior provides a direct visualization of how structural defects drive long‐range chemical patterning in nanoalloys. By acting as a kinetic conduit for periodic solute flux, the TB facilitates a transition to a state of internal heterogeneity that fundamentally diverges from the surface enrichment predicted by classical equilibrium segregation models.

The in situ HRTEM observations of TB‐mediated Au segregation are further supported by atomic‐resolution HAADF‐STEM imaging, which provides direct *Z*‐contrast evidence of the evolving compositional landscape. Figure 2F resolves two adjacent twin domains separated by a coherent {111} TB. The mirrored lattice symmetry and relatively uniform column intensities confirm that the domains initially exist as a homogeneous Cu(Au) solid solution. By contrast, Figure 2G captures the onset of Au segregation at the TB: a distinctly brighter atomic column emerges along the TB plane, marking the initial stage of compositional partitioning. The corresponding intensity line profiles taken across the dotted boxes show a pronounced intensity peak at the twin interface, directly indicating Au enrichment at the atomic site. These HAADF‐STEM results corroborate the time‐resolved HRTEM observations and further confirm the TB as the energetically preferred nucleation site for the development of Au‐rich lamellae.

Following extended vacuum annealing (∼1 h at 450°C), the NPs develop well‐defined, chemically separated twin lamellae (Figure 2H). The corresponding FFTs (insets) reveal distinct lattice parameter shifts between the domains: the Au‐rich domain exhibits expanded interplanar spacing—0.20 nm for {200} and 0.22 nm for {111}—compared with 0.18 and 0.20 nm, respectively, in the Cu‐rich domain. This lattice expansion follows Vegard's law: higher Au content expands the local lattice because Au has a larger lattice constant (0.402 nm) than Cu (0.361 nm). Despite this enrichment, no superlattice reflections in the FFT of the Au‐rich lamella, confirming that the alloy remains a chemically disordered solid solution. This is consistent with the experimental temperature (∼450°C), which exceeds the critical ordering–disordering transition temperature (∼390°C) for the Cu–Au system, thus precluding long‐range intermetallic.

### 2.3 AP‐XPS Measurements

To corroborate the structural and compositional evolution observed via in situ TEM and STEM, we perform AP‐XPS measurements on Cu_75_Au_25_ NPs supported on highly oriented pyrolytic graphite (HOPG). The evolution of the Au 4f, Cu 3p, and Cu LMM regions is monitored sequentially during annealing. For the Au 4f and Cu 3p spectra, a relatively low photon energy (650 eV) is employed. This choice enhances surface sensitivity by reducing the inelastic mean free path of the emitted photoelectrons and enables simultaneous acquisition of the Au 4f and Cu 3p signals within a single spectral window. Such simultaneity is critical for the quantification of the surface alloy composition. By contrast, Cu 2p spectra, typically probed at higher photon energies, are less surface‐sensitive and do not allow concurrent Au detection, thereby limiting precise stoichiometric tracking. Figure 3A shows the Au 4f and Cu 3p spectra collected during stepwise annealing from room temperature to ∼600°C under ultrahigh vacuum (UHV)—conditions mirroring the thermal protocols of our in situ TEM experiments. Binding energies (BEs) are calibrated to the C 1s peak (284.8 eV) of the HOPG support and the Cu 2p_3/2_ peak (932.6 eV) [58].

**FIGURE 3:**
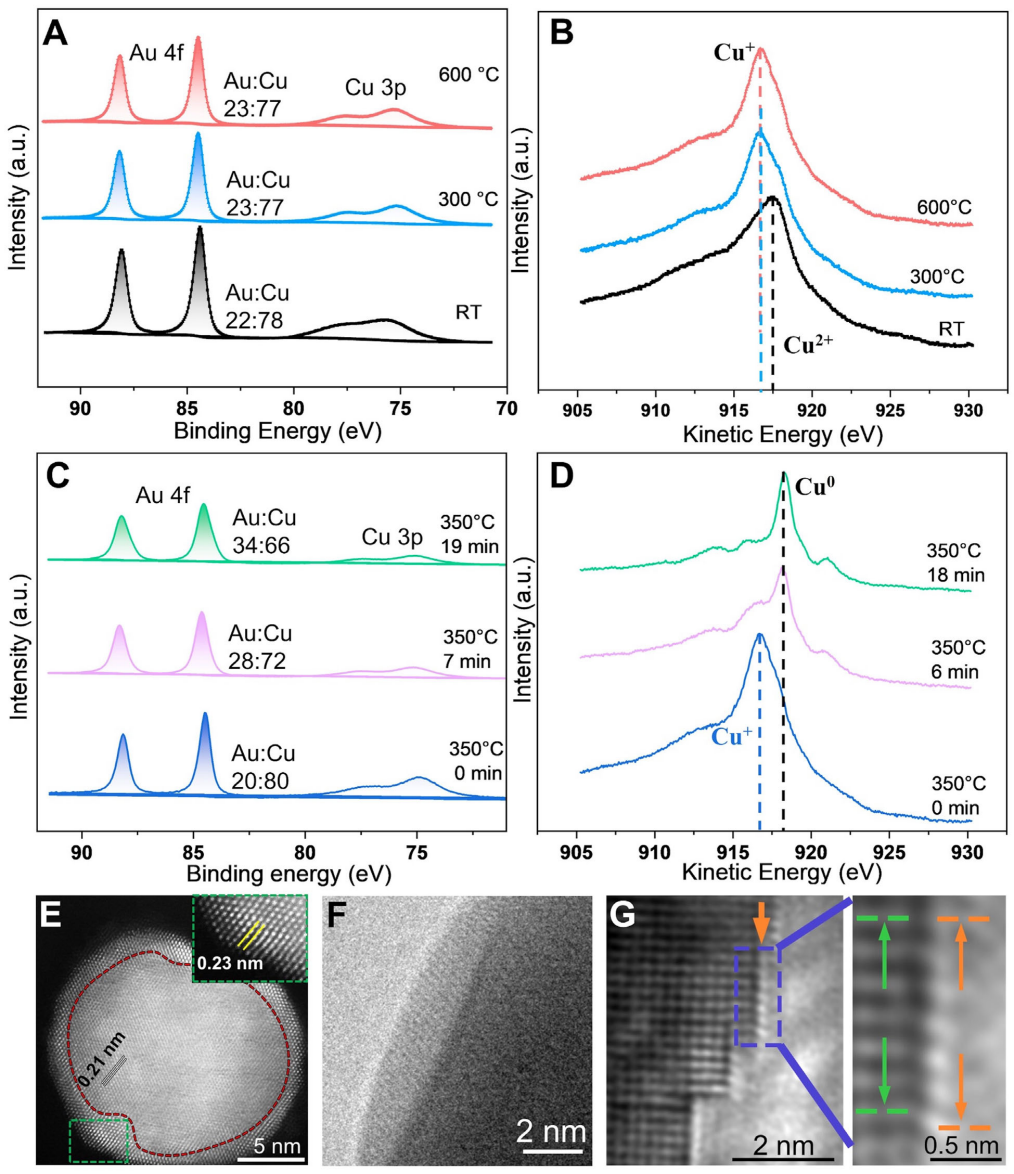
Environmental control of segregation pathway in Cu_75_Au_25_ NPs. (A,B) Time‐resolved Au 4f, Cu 3p, and Cu LMM spectra during UHV annealing up to 600°C, showing the persistence of the CuO*_x_* shell and the absence of Au surface segregation. (C,D) Spectroscopic evolution during annealing at 350°C under 1 Torr H_2_; the complete reduction of CuO*_x_* to metallic Cu triggers progressive Au surface enrichment, evidenced by the increasing Au/Cu intensity ratio. Au 4f and Cu 3p spectra in (A,C) are collected using a photon energy of 650 eV, and Cu LMM spectra using 1150 eV to maintain surface sensitivity. (E) Ex situ HAADF‐STEM image of an H_2_‐annealed NP, resolving a distinct Au‐rich shell (∼1 nm). The inset (upper‐right) provides a magnified view of the region marked by the green dashed box, revealing an increased surface lattice spacing (∼0.23 nm) relative to the bulk (∼0.21 nm). (F) In situ TEM image showing the local removal of the native CuO*_x_* via converged electron‐beam sputtering at 450°C. (G) HRTEM image along the [100] zone axis of the sputtered region. The magnified view resolves an Au‐rich outermost atomic layer characterized by an expanded lattice spacing relative to the underlying Cu(Au) lattice. The orange and green arrows delineate an identical number of atomic columns in the surface and subsurface layers, respectively; the greater lateral span of the surface arrows quantitatively confirms the expansion of the surface lattice spacing. The observation demonstrates that Au readily occupies the surface once the oxide‐induced kinetic barrier is eliminated, restoring the classical surface‐segregation pathway.

The Au 4f region exhibits a well‐resolved doublet (Au 4f_5/2_ and Au 4f_7/2_), with the Au 4f_7/2_ component consistently positioned at 84.5 eV. This BE is positively shifted relative to metallic Au (at 84.0 eV), a characteristic signature of Au in a chemically alloyed Cu–Au environment [31, 32, 33, 35, 59]. The persistence of this shift throughout the annealing process indicates that Au remains stabilized within the alloy lattice, and crucially, does not segregate to the surface under UHV annealing. Simultaneously, the Cu 3p_3/2_ peak, located at BE = 75.1 eV, reflects contributions from metallic Cu and a Cu_2_O phase [31, 60, 61], consistent with the native oxide observed in HAADF‐STEM.

To quantitatively track surface composition evolution during annealing, the integrated Au 4f/Cu 3p peak area ratio is normalized by their Scofield scattering factors (*σ*_Cu3p_ = 2.478, *σ*_Au4f_ = 17.12). The initial Au/Cu ratio is determined to be 0.28, which is lower than the nominal bulk ratio of 0.33. This initial deficiency confirms the presence of a Cu‐rich surface overlayer, primarily composed of native CuO*_x_*, which effectively passivates the NP surface and dictates the initial segregation landscape.

During UHV annealing, the spectral shapes remain essentially unchanged, and the Au/Cu ratio stays nearly constant, demonstrating that Au does not migrate to the surface in the absence of a reducing gas environment. The persistence of the native CuO*_x_* overlayer, which remains unreduced under UHV conditions, imposes a kinetic barrier that suppresses classical surface segregation. These results directly substantiate the TEM observations in Figures 1 and 2: by blocking the pathway to the exterior, the oxide shell redirects Au migration toward internal lattice defects, specifically coherent TBs, thereby catalyzing the formation of Au‐rich and Cu‐rich lamellae inside the NPs.

Coordinated Cu LMM Auger spectra (Figure 3B) are acquired to further elucidate the redox state of the surface. Although Cu 2p and 3p photoelectron peaks are commonly used for element identification, they are insufficient for distinguishing between Cu^0^ and Cu^+^ species because their chemical shifts are very small and often masked by peak broadening. By contrast, the Cu LMM Auger lines exhibit distinct kinetic energy differences, enabling unambiguous discrimination between metallic and ionic Cu species. In the as‐prepared NPs, the dominant Cu LMM kinetic energy is centered at ∼917.9 eV, characteristic of Cu^2+^ in CuO [58]. Annealing at ∼300°C under UHV induces a shift to 916.9 eV (Cu^+^), indicating a partial reduction of the CuO overlayer to Cu_2_O. Importantly, this Cu^+^ signature persists even after annealing at ∼600°C, confirming the thermal stability of the Cu_2_O phase under vacuum. The retention of this oxide shell throughout the thermal annealing ensures that Au surface enrichment remains energetically and kinetically hindered, consistent with the steady Au/Cu ratio observed in Figure 3A.

In stark contrast, in situ reduction under 1 Torr H_2_ at 350°C triggers a fundamental shift in the segregation landscape (Figure 3C). The Au 4f intensity increases steadily relative to the Cu 3p signal, reflecting the progressive removal of surface CuO*_x_* and the consequent emergence of Au at the surface. As the oxide is reduced, the Au/Cu area ratio rises and saturates at ∼0.50, marking the transition to a steady‐state Au surface segregation level. Although the Cu 3p spectra show no discernible core‐level shifts—owing to the close binding energies between Cu^0^ and Cu^+^ in the Cu 3p region—the overall Cu 3p intensity attenuates with continued H_2_ annealing. This attenuation arises from Au surface segregation, which increasingly screens the underlying Cu signal. Complementary Cu LMM Auger spectra (Figure 3D) further confirm that Cu^+^ is fully reduced to metallic Cu^0^ (918.6 eV) under these H_2_ annealing conditions.

Collectively, the evolution of the Au 4f, Cu 3p, and Cu LMM signals demonstrates that the chemical state of the surface dictates the compositional segregation trajectory of the alloys. H_2_ exposure effectively “unlocks” the surface, enabling extensive Au surface segregation. Conversely, under UHV, the persistent CuO*_x_* shell acts as a chemical seal, forcing Au atoms to redistribute internally along TBs. This spectroscopic evidence, along with the TEM observations, provides a bridge between the macroscopic environment and the observed atomistic patterning within the nanoalloy interior.

Ex situ HAADF‐STEM imaging of H_2_‐annealed Cu_75_Au_25_ NPs further provides a direct structural comparison to the UHV‐annealed samples, confirming the transition to a classical surface segregation regime (Figure 3E). Following H_2_ treatment, the NPs exhibit a well‐defined core–shell architecture, characterized by a high‐intensity (∼1 nm) exterior layer. This *Z*‐contrast enhancement at the surface corresponds to an Au‐enriched shell, which also displays a dilated lattice spacing relative to the core—a direct result of incorporating the larger Au atoms.

Notably, the absence of a low‐contrast outer layer confirms complete reduction of the CuO*_x_* shell, in contrast to the as‐prepared NPs in Figure 1B. Furthermore, the particle core remains chemically homogeneous, showing no evidence of the twinning or lamellar compositional modulation observed under UHV annealing (Figures 1 and 2). This bifurcation in structural trajectory demonstrates that the internal, twin‐mediated chemical separation is contingent upon the presence of the surface oxide; once the oxide barrier is eliminated, the system reverts to its thermodynamic preference for Au surface enrichment, effectively quenching the driving force for internal patterning.

The kinetics of Au surface segregation under oxide‐free conditions are further investigated using in situ HRTEM. To simulate a pristine metallic environment within the TEM, the native CuO*_x_* layer is locally removed through controlled electron‐beam sputtering at 450°C for ∼30 s using a highly converged probe. The resulting surface (Figure 3F) exhibits a characteristic brightness contrast arising from localized beam‐induced thinning. Figure 3G presents the corresponding atomic‐resolution image along the [100] zone axis, revealing an Au‐rich outermost atomic layer. The enlarged view quantifies a dilated lattice spacing of the surface layer relative to the subsurface lattice—a structural signature of Au enrichment. These observations confirm that Au atoms readily segregate to the surface once the CuO*_x_* barrier is eliminated, a process that effectively precludes the twin‐mediated internal compositional patterning.

Although direct atomic‐resolution elemental mapping of individual Au and Cu atomic columns is beyond the capability of the current EDS instrumentation, the proposed oxidation‐induced intragranular chemical redistribution is supported by multiple complementary experimental observations. The EDS line‐scan analysis across the twin lamellae (Figure 1G) reveals spatial oscillations in the Au and Cu signals, while the corresponding atomic‐resolution HAADF‐STEM images (Figure 1H) exhibit enhanced *Z*‐contrast intensity along the twin boundaries, consistent with preferential enrichment of the heavier Au species at these internal interfaces. Furthermore, the AP‐XPS measurements demonstrate that the surface Au/Cu ratio remains suppressed in the presence of the surface CuO*_x_* layer, indicating that oxidation inhibits the conventional outward segregation of Au and redirects Au atoms from the surface toward the particle interior. Upon subsequent H_2_ reduction, removal of the CuO*_x_* surface layer restores the thermodynamic driving force for Au surface segregation, as evidenced by the increased surface Au/Cu ratio, the in situ HRTEM observation of expanded lattice spacing in the surface region relative to the subsurface region, and ex situ HAADF‐STEM imaging showing pronounced surface Au enrichment accompanied by the disappearance of the previously observed Au‐rich twin‐boundary lamellae. This reversible redistribution of Au between the particle surface and interior in response to the surface chemical state, together with the consistent structural and spectroscopic evidence, provides strong support for the oxidation‐induced intragranular chemical patterning mechanism proposed here.

It is important to distinguish the roles of temperature and surface chemistry in the observed elemental redistribution. Elevated temperature is essential for activating atomic diffusion; however, it is not the primary driving force for the oxidation‐induced internal chemical patterning. The in situ AP‐XPS measurements (Figure 3A,B) show that, in the absence of a reducing atmosphere, the CuO*_x_* surface layer remains stable from room temperature to 600°C, while the surface Au/Cu ratio remains consistently suppressed throughout this temperature range. Thus, thermal activation alone does not induce the conventional outward segregation of Au when the oxidized surface is maintained. By contrast, introducing H_2_ completely reduces the CuO*_x_* surface layer at ∼350°C, and this lower temperature is already sufficient to restore Au surface segregation, as evidenced by the progressive increase in the surface Au/Cu ratio (Figure 3C,D). The fact that Au surface enrichment occurs at 350°C after oxide removal, whereas no measurable Au surface segregation is observed even at 600°C in the presence of the surface oxide, demonstrates that the decisive factor is the surface chemical state rather than the annealing temperature. Therefore, temperature primarily provides the kinetic activation required for atomic migration, whereas the surface oxide determines the thermodynamic driving force and, consequently, the direction of Au transport. The observed internal chemical patterning thus arises from the interplay between thermally activated diffusion and oxidation‐induced reversal of the segregation pathway, rather than from thermal annealing alone.

### 2.4 DFT Modeling

To elucidate the atomistic mechanisms by which TBs facilitate long‐range compositional patterning and promote the formation of alternating Au‐rich and Cu‐rich lamellae, DFT calculations are performed to assess both the stability of Au segregation to the TB and the energetics associated with inter‐domain Au transfer. Guided by our HAADF‐STEM observations (Figures 1 and 2), we model a twinned Cu(Au) solid solution and monitor the system energy as Au atoms are sequentially relocated from the bulk lattice to the {111} TB interface (Figure 4A). As the boundary occupancy increases, the local environment transitions from dilute Au monomers to Cu–Au pairwise configurations, and eventually to Au–Au nearest‐neighbor contacts at high coverage.

**FIGURE 4:**
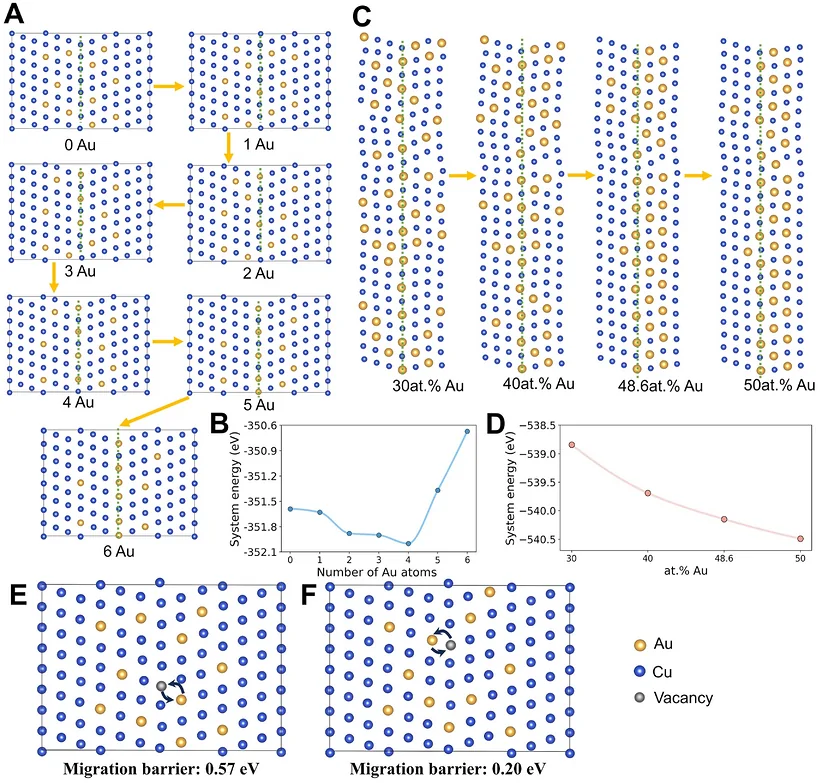
DFT analysis of the thermodynamic driving forces for twinning‐mediated chemical separation. (A) Computational models illustrating sequential migration of Au solute atoms from the bulk lattice to the {111} TB. The simulation captures the evolution of the local chemical environment from dilute Au monomers to high‐density configurations, specifically highlighting the transition from isolated solutes to the Cu–Au pairwise arrangement, and finally to Au–Au nearest‐neighbor configurations once the boundary becomes overpopulated. (B) The corresponding DFT energies for the Au‐segregation configurations in (A), demonstrating monotonic thermodynamic stabilization up to the Cu–Au pairwise configuration (maximum at ∼50% occupancy), followed by a sharp energetic penalty upon the introduction of Au–Au nearest‐neighbor contacts. (C) Sequential transfer of Au atoms from the “donor” (left) twin domain to the “acceptor” (right) domain, simulated while maintaining the optimized Cu–Au pairwise stoichiometry at the TB. As Au accumulates in the right domain, its local environment evolves from isolated Au atoms separated by Cu to the Cu–Au pairwise configuration. These models represent the “uphill” diffusion process required for lamellar growth. (D) DFT energies for the configurations in (C), showing progressive stabilization as the acceptor domain stoichiometry approaches ∼50 at.% Au. The energy minimum corresponds to a configuration of maximized Cu–Au coordination, providing a thermodynamic explanation for the formation of the Au‐rich lamellar architecture observed experimentally. (E,F) Climbing‐image nudged elastic band (CI‐NEB) calculations of the energy barriers for vacancy‐mediated Au migration from the twin‐lamella matrix to the TB, corresponding to first (E) and second (F) Au segregation events.

The calculated formation energies (Figure 4B) reveal a monotonic stabilization as the initial five Au atoms segregate to the TB, reaching a minimum at a stoichiometric Au/Cu ratio of 1. At this concentration, the TB adopts a maximally stabilized Cu–Au pairwise arrangement. However, further Au incorporation introduces unfavorable Au–Au bonds, resulting in a sharp energetic penalty. These results identify the TB as a robust thermodynamic sink for Au segregation with a critical saturation threshold at ∼50% occupancy. Beyond this limit, the boundary becomes destabilizing, providing a potent driving force to partition excess Au into the adjacent lattice rather than further enriching the interface.

To understand how this TB‐stabilized segregation propagates into the bulk to form alternating lamellae, we model the transfer of Au atoms from the left “donor” twin domain to the right “acceptor” domain while maintaining the energetically favored Cu–Au pairwise configuration at the boundary (Figure 4C). As Au accumulates in the right domain, its local environment evolves from isolated Au atoms to arrangements where Au atoms approach one another through Cu intermediates, and finally to the energetically preferred Cu–Au pairwise configuration.

The DFT energies (Figure 4D) decrease monotonically as more Au atoms are transferred into the right domain, reaching maximal stabilization when the domain attains an ∼50% Au concentration configured in Cu–Au pairs. Notably, this DFT‐predicted optimal composition agrees well with the experimentally measured Au content (∼46 at.%) in the Au‐rich lamellae (Figure 1G). Together, these results show that the energetic preference for optimized Cu–Au pairwise arrangements—both along the TB and within the adjacent lattice—provides a unified thermodynamic origin for the observed bulk chemical separation. TBs act as initial sinks for Au enrichment and, once saturated, drive coordinated solute redistribution that gives rise to the alternating Cu‐rich and Au‐rich lamellae of the chemically separated state within the NP interior.

To clarify the kinetics of TB‐induced Au redistribution, climbing‐image nudged elastic band (CI‐NEB) calculations are performed to determine the migration pathway and activation energy for Au diffusion from the Cu–Au matrix to the TB. The first Au segregation event proceeds through a vacancy‐mediated exchange mechanism, where an Au atom migrates from a bulk lattice site to a pristine TB site with an activation barrier of 0.57 eV (Figure 4E). The moderate barrier indicates that the TB provides a structurally favorable environment for accommodating Au segregation. After the initial Au incorporation, the migration barrier for a second Au atom decreases to 0.20 eV (Figure 4F), owing to the local lattice expansion and structural relaxation induced by the presegregated Au atom. This cooperative effect facilitates further Au enrichment at the TB. Combined with the lower formation energies associated with Au‐rich TB configurations, these results demonstrate that TB‐mediated Au segregation is both thermodynamically favored and kinetically accessible, enabling oxidation‐driven internal chemical patterning in Cu–Au nanoalloys

### 2.5 Impact of Internal Chemical Patterning on Catalysis

Alloying remains a cornerstone of catalyst design, as synergistic electronic and geometric interactions between constituent metals often yield activities that surpass monometallic limits. However, the thermodynamic stability of these active sites is frequently compromised by dynamic restructuring under reaction conditions. Cu(Au) NPs exemplify this challenge; while effective for CO oxidation [34, 62], methanol oxidation [63], steam reforming [64], and the water–gas shift reaction [64], their efficacy is predicated on the maintenance of surface Cu–Au ensembles. Our findings establish a critical governing principle: the presence of a surface oxide layer acts as a “chemical seal” that inhibits Au surface segregation, instead redirecting solute flux toward internal TBs. This internal partitioning into Au‐rich and Cu‐rich lamellae fundamentally alters the surface‐to‐bulk stoichiometry, directly impacting the availability of synergistic active sites.

To evaluate the catalytic consequence of this internal redistribution, CO conversion profiles (Figure 5A) are recorded during CO oxidation catalyzed by Cu_75_Au_25_ NPs subjected to four pretreatment conditions designed to modify their internal and surface configurations: (State I) as‐prepared (uniform solid‐solution); (State II) He‐annealed at 450°C (structural twinning and internal lamellar segregation); (State III) He annealing followed by O_2_ annealing at 260°C (CuO*_x_* shell atop internal lamellar segregation); (State IV) He + O_2_ annealing followed by H_2_ annealing at 400°C (restoration of a metallic surface via oxide removal, resulting in extensive Au surface segregation).

**FIGURE 5:**
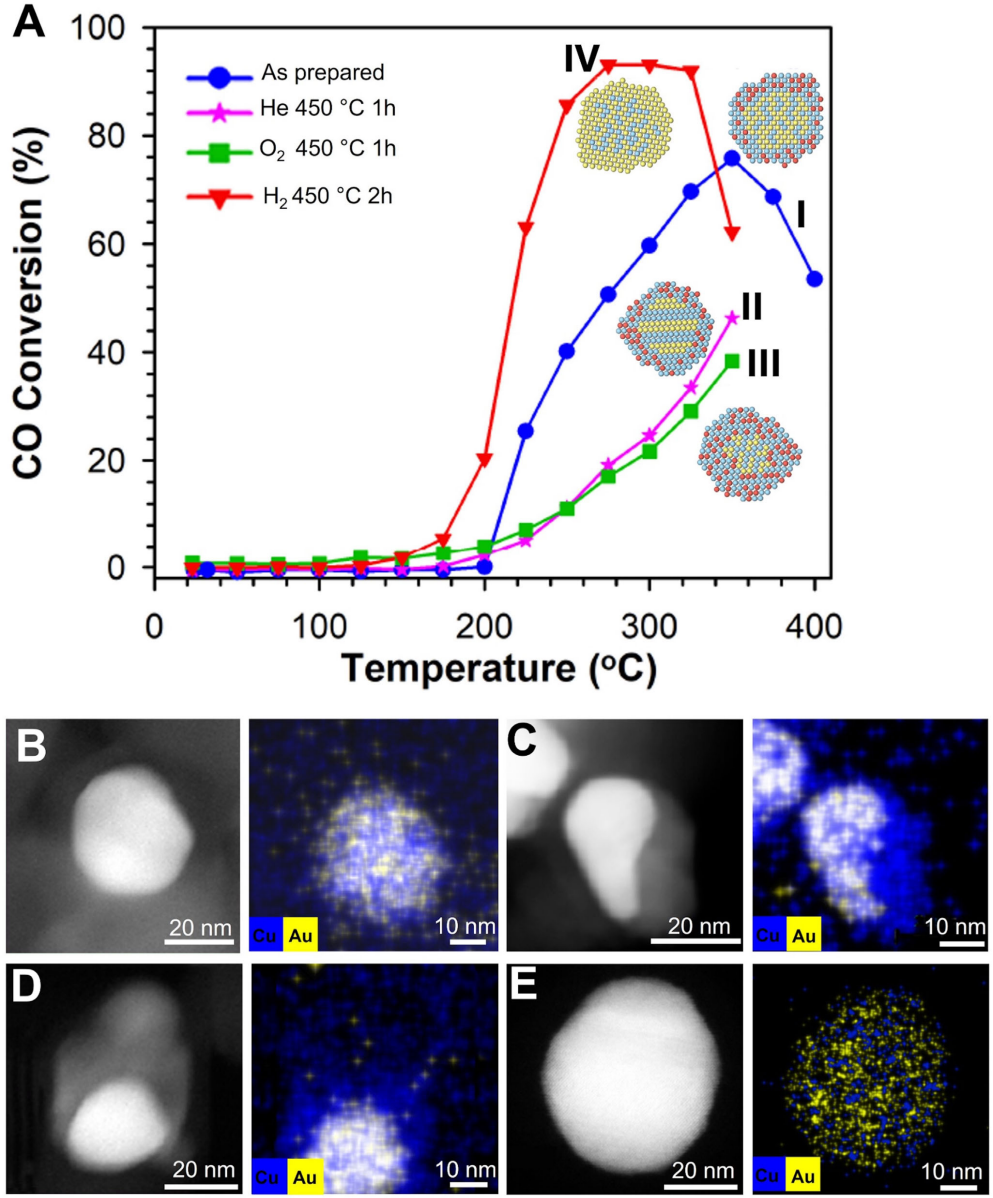
Correlation between compositional partitioning and catalytic CO oxidation performance. (A) CO conversion profiles (CO + ½O_2_ = CO_2_) as a function of temperature for Cu_75_Au_25_ NPs across four distinct structural states: (I) as‐prepared (uniform solid solution); (II) He annealed at 450°C (internal lamellar separation mediated by structural twinning); (III) He annealed followed by O_2_ annealing at 260°C (surface CuO*_x_* formation with retained internal compositional partitioning); (IV) He + O_2_ annealed followed by H_2_ annealing at 400°C (oxide reduction and restoration of Au surface segregation). The highest conversion efficiency and shift toward lower activation temperature in State IV highlight the superior activity of the Au‐enriched surface compared to the Au‐depleted surfaces in States II and III. (B) Postreaction HAADF‐STEM images and the corresponding EDS maps characterizing the structural stability of the four catalysts after CO oxidation tests: (B) Sample I; (C) Sample II; (D) Sample III; and (E) Sample IV.

The catalytic performance correlates strongly with these structural configurations. Samples II and III show markedly reduced CO conversion relative to the as‐prepared NPs (State I). In State II, the defect‐mediated migration of Au atoms to internal TBs leaves the surface Cu‐rich and deficient in Au, effectively “diluting” the mixed Cu–Au active ensembles. In State III, the additional O_2_ annealing grows a CuO*_x_* shell that further passivates the surface and reinforces the internal sequestration of Au. In both instances, the partitioning of Au into the NP interior—directed by the combination of surface oxidation and structural twinning—prevents the assembly of mixed Cu–Au active sites required for efficient CO conversion.

By contrast, the H_2_‐pretreated NPs (State IV) display the highest catalytic activity, achieving peak CO conversion at a substantially lower temperature than all other configurations. As demonstrated by AP‐XPS and STEM analysis (Figure 3), H_2_ annealing effectively removes surface CuO*_x_*, restores a pristine metal surface, and enables extensive Au surface segregation. The resulting Au‐rich shell hosts abundant Au–Cu surface sites that facilitate CO activation and O_2_ dissociation. These catalytic trends confirm that performance is intrinsically tied to the surface accessibility of Au. Structural trajectories characterized by surface oxidation or internal defect‐mediated trapping (States II and III) result in poor efficacy due to the depletion of Cu–Au surface ensembles. Conversely, restoring the pathway for Au surface segregation (State IV) optimizes the surface chemistry by bypassing the internal Au sequestration at TBs.

Postcatalytic STEM analysis (Figure 5B–E) resolves the long‐term stability of these environment‐dependent structural pathways. State I (as‐prepared) retains a largely uniform composition after reaction, consistent with its initially homogeneous alloy state. Without significant oxide barriers, modest Au surface segregation can occur during reaction, enabling moderate catalytic performance. State II (He‐annealed) continues to exhibit pronounced internal lamellar segregation with alternating Cu‐rich and Au‐rich regions. This segregation remains stable under reaction conditions, indicating that once Au becomes trapped along TBs, catalytic heating is insufficient to overcome the energetic barrier for redistribution. The persistent Cu‐rich surface explains its lower catalytic activity. State III (He + O_2_ annealed) shows a thick Cu‐rich (CuO*_x_*‐derived) shell and strong Au enrichment in the core. This configuration reflects the thermodynamic preference of Au to avoid the oxidized surface region, driving it further into the interior during reaction. The retained CuO*_x_* shell and Au‐depleted surface correlate with its poorest catalytic activity. State IV (H_2_‐treated) displays the most uniformly distributed Au among all samples after reaction. The removal of CuO*_x_* before catalysis allows Au to segregate to the surface and remain mobile enough during reaction to maintain a stable Au‐enriched shell. This persistent surface Au population maps directly onto its superior low‐temperature activity.

Taken together, the catalytic performance and postreaction morphologies converge on a coherent mechanistic picture: surface accessibility of Au—not merely overall alloy composition—dictates activity. States that trap Au inside the NPs (II and III) show both internal segregation and poor activity, while states that allow Au to reach and remain at the surface (I and IV) show higher activity, with state IV achieving the optimal surface configuration. By identifying the surface oxide as the decisive “gatekeeper” that directs Au flux, we establish a structural roadmap for maintaining active surface ensembles in bimetallic oxidation catalysts.

Although the catalytic measurements demonstrate that oxidation‐induced structural evolution influences the catalytic behavior of Cu–Au nanoalloys, the individual contributions of the surface oxide layer and the internal Au‐rich lamellar segregation cannot be quantitatively decoupled in the present study because these two structural features are intrinsically coupled through the same oxidation‐driven segregation process. Rather than representing independent catalytic descriptors, they constitute two interconnected manifestations of the environment‐induced chemical restructuring of the alloy nanoparticles. Our combined AP‐XPS, in situ HRTEM, and ex situ HAADF‐STEM results (Figure 3) reveal that this restructuring is highly dynamic and reversible. Upon H_2_ treatment, removal of the surface oxide restores the thermodynamic preference for Au surface segregation, resulting in pronounced Au enrichment at the particle surface and the absence of the previously observed Au‐rich twin‐boundary lamellae. This reversible redistribution of Au between the surface and particle interior demonstrates that the catalyst architecture continuously adapts to the surrounding chemical environment. Therefore, the observed catalytic behavior should be considered as a consequence of the coupled evolution of surface chemistry and internal elemental distribution under reactive conditions, rather than a static contribution from either the surface oxide or internal chemical patterning alone. Quantitatively resolving the individual roles of these structural motifs will require future studies using independently tunable model catalysts and operando characterization approaches.

## 3 Discussion

The interplay between external chemical environments and structural defects often leads to unconventional mass transport phenomena. Similar to how NP‐support interfaces can dictate distinct oxidation dynamics through epitaxial matching [65], our results demonstrate that internal twinning defects act as regulatory sites that reshape the energy landscape during oxidation. This study reveals a previously unrecognized mechanism of twinning‐assisted internal chemical separation, highlighting the profound influence of surface states on the structural trajectory of nanoalloys. Traditionally, chemical segregation in bimetallic NPs is viewed through the lens of surface energy minimization, typically yielding core–shell architectures in which the component with lower surface energy (i.e., Au) preferentially enriches the exterior. This configuration maximizes Cu–Au synergy, enhancing catalytic activity, stability, and selectivity. However, our results demonstrate that surface oxidation can invert this classical segregation behavior. By forming a CuO*_x_* overlayer, the NP surface suppresses conventional Au enrichment and instead redirects Au flux into the particle interior along TBs, generating alternating Au‐rich and Cu‐rich lamellae. This oxidation‐ and defect‐driven internal compositional partitioning contrasts with prior models of surface‐dominated chemical separation and demonstrates the intricate interplay between surface chemistry and internal defect structures in dictating elemental redistributions.

The nucleation of twin lamellae is closely coupled to the oxidation‐induced redistribution of Au and the thermally activated mechanical response of the Cu–Au alloy. Elevated temperature provides the kinetic activation required for Au diffusion, allowing local Au concentration fluctuations to develop within the Cu–Au solid solution as oxygen chemisorption drives Au away from the surface into the particle interior. Owing to the atomic size mismatch between Au and Cu, these compositional fluctuations generate localized lattice distortions and associated shear stresses. When the local resolved shear stress exceeds the critical threshold for partial dislocation emission, twinning becomes an energetically favorable mechanism for accommodating the local strain. This interpretation is consistent with previous molecular dynamics simulations and generalized stacking‐fault calculations of Cu–Au alloys, [8] which showed that the critical resolved shear stress for partial dislocation motion in Cu at 350°C is only ∼11 MPa (corresponding to a shear strain of ∼0.01%) and that Au alloying produces little change in the unstable stacking‐fault energy of Cu, indicating that twinning can be readily activated by relatively small composition‐induced local strain fluctuations under the present experimental conditions. Once TBs are formed, they serve as energetically favorable sinks for the redistributed Au. Our DFT calculations show that the formation energy decreases monotonically with increasing Au concentration at the TB and reaches a minimum for a local composition approaching CuAu, demonstrating that Au enrichment stabilizes the TBs through favorable Cu–Au heteroatomic bonding. Therefore, twin formation and Au segregation constitute a cooperative self‐organization process: elevated temperature enables the compositional fluctuations necessary for twin nucleation, the oxidized surface redirects Au into the particle interior by reversing the preferred segregation pathway, and the newly formed TBs subsequently capture and stabilize the redistributed Au, giving rise to the observed internal Au‐rich lamellar chemical patterning.

The formation of the internal Au‐rich lamellar pattern arises from the interplay between the intrinsic thermodynamics of the Cu–Au alloy and oxidation‐induced modification of the Au segregation pathway. Because the Cu–Au system possesses a negative enthalpy of mixing (Δ*H*_mix_ < 0), Cu–Au heteroatomic bonding is energetically favored over Cu–Cu and Au–Au bonding, driving the alloy toward configurations that maximize heteroatomic nearest‐neighbor interactions. Our DFT calculations (Figure 4) show that this thermodynamic driving force is realized through progressive Au enrichment at TBs, where the formation energy decreases monotonically with increasing Au concentration and reaches a minimum for a local composition approaching CuAu. Consequently, the twin lamellae constitute energetically favorable sinks that simultaneously minimize the twin‐boundary energy and maximize Cu–Au heteroatomic bonding. Under oxide‐free conditions, however, this equilibrium tendency is largely obscured because Au preferentially segregates to the free surface owing to its lower surface energy.

Surface oxidation fundamentally redirects this segregation pathway. Additional DFT calculations using a Cu_90_Au_10_(111) slab model (Figure S4) show that, in the absence of oxygen adsorption, Au surface segregation is favored by 1.30 eV relative to a Cu‐terminated surface. Upon adsorption of 0.5 monolayer oxygen, however, the energetic ordering is completely reversed, with the Cu‐terminated surface becoming 1.04 eV more stable. These results demonstrate that oxygen chemisorption stabilizes Cu at the outermost surface while driving Au into the subsurface and particle interior, thereby reversing the preferred segregation direction from Au‐outward to Cu‐outward/Au‐inward. In this sense, the oxidized surface acts as a chemical pump that redirects Au away from its equilibrium surface segregation pathway without altering the fundamental thermodynamic driving force for alloy ordering. The resulting inward Au flux is subsequently captured by twin boundaries, where the local composition evolves toward the energetically preferred CuAu stoichiometry.

This mechanism is independently supported by the AP‐XPS measurements (Figure 3), which show that the surface Au/Cu ratio remains suppressed while the CuO*_x_* shell is present but increases progressively following reduction of the oxide shell, demonstrating that surface oxidation inhibits the conventional Au surface segregation and instead redirects Au into the particle interior. Consistent with this interpretation, previous in situ TEM studies of Cu–Au alloys [7, 8] showed that Au segregates to the oxide‐free surface under H_2_, whereas oxygen chemisorption induces Cu surface segregation and displaces Au from the surface. These studies further demonstrated that oxygen chemisorption alone is sufficient to reverse the segregation direction without forming bulk Cu_2_O, in excellent agreement with our DFT calculations and AP‐XPS observations. Collectively, these results show that the observed internal chemical patterning is not driven by surface oxidation itself. Rather, it results from the intrinsic thermodynamic tendency of the alloy to maximize Cu–Au heteroatomic bonding, while surface oxidation redirects the preferred Au segregation pathway from the free surface to the particle interior, thereby enabling the alloy to evolve toward a lower‐energy internally ordered configuration.

The observed uphill diffusion of Au from Au‐poor to Au‐rich lamellae is particularly noteworthy, as it departs from the classical Fickian expectation that atoms migrate down their concentration gradients. This counterintuitive mass transport arises from the synergistic coupling between surface oxidation and the internal thermodynamic landscape of the NPs. The formation of a CuO*_x_* overlayer effectively passivates the surface, suppressing the Au surface segregation that would otherwise occur on a pristine alloy surface. Consequently, Au redistribution is no longer governed by surface segregation but by the bulk free‐energy landscape of the alloy. Within the NP interior, the negative enthalpy of mixing favors Cu–Au heteroatomic bonding over homoatomic interactions. This thermodynamic preference drives Au atoms to migrate from the Au‐poor lamellae toward the Au‐rich lamellae, thereby increasing the number of energetically favorable Cu–Au pairwise interactions and lowering the bulk mixing free energy. As a result, oxidation‐induced suppression of surface segregation redirects atomic transport into an uphill diffusion pathway that reduces the overall free energy of the NPs despite the opposing Au concentration gradient.

TBs play a pivotal role in this internal Au redistribution by locally modifying lattice strain and atomic coordination, thereby creating energetically favorable pathways for Au migration. Our EDS measurements (Figure 1G) and DFT calculations (Figure 4) confirm that these defect sites act as preferential sinks for Au atoms, enabling enrichment up to ∼50 at.%, within the lamellar regions. This composition corresponds closely to the maximization of Cu–Au heteroatomic bonding; any further Au accumulation would lead to a higher degree of homoatomic Au–Au coordination, thereby reducing thermodynamic stability. DFT simulations confirm that this twin‐assisted segregation pathway is energetically downhill, providing the mechanistic route for the formation of the observed compositional lamellae. Taken together, these results reveal that surface oxidation acts as a chemical pump, redirecting Au flux away from the surface and toward defect‐mediated internal compositional ordering. Through the cooperative interplay of surface oxidation, alloy thermodynamics, and lattice defects, the system evolves toward a lamellar structure that maximizes heteroatomic bonding while suppressing conventional surface‐driven segregation.

From a catalytic perspective, these results carry significant implications. The disruption of surface Au enrichment by surface oxidation diminishes the formation of highly active Cu–Au surface ensembles, directly impacting catalytic performance in reactions such as CO oxidation, CO_2_ reduction, and selective hydrogenation. Our work establishes a clear connection between surface oxidation, internal chemical patterning, and catalytic degradation, offering a mechanistic framework to predict when and how nanoalloy catalysts may lose activity under reactive conditions.

Furthermore, these findings bridge a critical knowledge gap in nanoalloy science: the interplay between surface‐mediated chemical activity and bulk defect dynamics. Previous studies have emphasized equilibrium‐driven surface segregation [66] or oxidation‐induced core–shell transformations [67], yet our results demonstrate that dynamic redox environments and internal structural defects can fundamentally reconfigure internal compositional patterning. Recognizing and leveraging this interplay provides new opportunities for designing oxidation‐tolerant, high‐performance catalysts, as well as multifunctional NPs with controlled internal architectures.

The oxidation‐driven elemental redistribution mechanism proposed here is rooted in two general physical principles: (i) adsorbate‐induced reversal of surface segregation and (ii) chemically driven solute partitioning to energetically favorable internal interfaces. Both phenomena have been reported across a wide range of alloy systems, suggesting that the present mechanism is not unique to Cu–Au alloys. For example, DFT calculations by Svenum et al. showed that although Ag segregates to the surface of Pd_3_Ag(111) in vacuum, adsorption of either CO or O reverses the segregation direction and drives Pd to the surface [68]. Similarly, Dhouib and Guesmi demonstrated oxygen‐induced reversal of surface segregation in PdAu, NiAu, and PtAu alloys [69]. More fundamentally, He et al.  [70] demonstrated through atomic‐resolution microscopy and first‐principles calculations that segregation at coherent twin boundaries is governed predominantly by chemical bonding (electronic structure and coordination) rather than elastic strain minimization, establishing chemical bonding as the dominant thermodynamic factor controlling solute partitioning to coherent interfaces. This mechanism closely parallels the present Cu–Au system, where our DFT calculations show that progressive Au enrichment at the twin boundary continuously lowers the formation energy by maximizing favorable Cu–Au heteroatomic bonding. These observations suggest that the proposed chemical‐pump mechanism should be generally applicable to alloy systems exhibiting adsorbate‐induced segregation reversal together with energetically favorable internal interfaces capable of stabilizing the redistributed solute through favorable chemical bonding.

## 4 Conclusions

In summary, this work reveals an oxidation‐ and defect‐mediated pathway for internal chemical redistribution in Cu–Au NPs, establishing the fundamental mechanism for environment‐driven nanoalloy reconstruction beyond the conventional core–shell segregation paradigm. By combining in situ imaging, operando AP‐XPS measurements, and first‐principles calculations, we demonstrate that surface oxidation reverses the preferred Au surface segregation pathway, driving Au into the particle interior where TBs act as energetically favorable sinks for Au accumulation. The resulting internal Au‐rich/Cu‐rich lamellar patterning arises from the cooperative interplay between reactive surface chemistry, thermally activated atomic diffusion, and crystallographic defects. DFT calculations reveal that Au enrichment at TBs is stabilized by favorable Cu–Au heteroatomic bonding, while vacancy‐mediated migration pathways enable kinetically accessible Au redistribution. These findings establish a defect‐enabled chemical separation mechanism in bimetallic NPs that differs fundamentally from conventional surface‐driven segregation. More broadly, this work highlights how reactive environments can be harnessed to engineer internal chemical architectures in nanoalloys by coupling surface chemistry with defect‐mediated mass transport. The demonstrated relationship between oxidation, segregation reversal, and interface stabilization provides mechanistic insights for designing dynamically adaptive nanomaterials with improved performance and stability in catalytic, energy, and functional applications.

## 5 Materials and Methods

Cu–Au NPs were synthesized using a wet‐chemistry approach [71]. In situ vacuum annealing was performed using a Gatan double‐tilt heating holder in an FEI Titan 80–300 environmental TEM operated at 300 kV. STEM imaging and EDS line scans were acquired on an FEI Talos F200X microscope equipped with an X‐FEG electron source and a Super‐X EDS detector, operated at 200 kV. HAADF‐STEM images were acquired using a convergence semiangle of 22 mrad, a detector inner collection angle of 68 mrad, and a detector outer collection angle of 280 mrad. AP‐XPS measurements were carried out at the IOS beamline of the National Synchrotron Light Source II (NSLS‐II), Brookhaven National Laboratory. DFT calculations were performed using the Vienna Ab initio Simulation Package (VASP). Additional experimental and computational details are provided in the supplementary information.

## Conflicts of Interest

The authors declare no conflicts of interest.

## Supporting information




**Supporting File 1**: smll75003‐sup‐0001‐SuppMat.docx.

**Supporting File 2**: smll75003‐sup‐0002‐SI1_video.avi.

**Supporting File 3**: smll75003‐sup‐0003‐SI2_video.avi.

## Data Availability

The data that supports the findings of this study are available in the supplementary material of this article.
