# Supplementary material for: Oxidation‐Induced Internal Chemical Patterning in Copper–Gold Nanoalloys
Source: Small. 2026 Aug 4;22(56):e75003. doi: 10.1002/smll.75003 (PMC13653352; doi:10.1002/smll.75003)
Supplement: Supplementary file 1 [file SMLL-22-e75003-s002.docx]

**Supplementary Information for**

**Oxidation-Induced Internal Chemical Patterning in Cu-Au Nanoalloys**

Zhikang Zhou^1^^†^, Hanlei Zhang^1†^, Dongxiang Wu^1^, Jianyu Wang^1^, Xiaobo Chen^1^, Chaoran Li^1^, Shan Yan^2^, Jing Li^2^, Adrian Hunt^3^, Iradwikanari Waluyo^3^, Chuanjian Zhong^2^, Guangwen Zhou^1^*

^1^Materials Science and Engineering Program & Department of Mechanical Engineering, State University of New York, Binghamton, New York 13902, United States

^2^Department of Chemistry, State University of New York, Binghamton, New York 13902, United States

^4^National Synchrotron Light Source II, Brookhaven National Laboratory, Upton, New York, 11973, United States

**This file includes:**

Materials and Methods

Table S1, S2

Figs. S1 to S3

Legends for supplementary videos S1 to S2

References

**Other supplementary materials for this manuscript include the following**

Supplementary Videos 1 to 2

**Materials and Methods**

**Synthesis of Cu(Au) NPs.** An aqueous solution containing ~2.0 × 10^-4^ M hydrogen tetrachloroaurate (HAuCl_4_, Aldrich, 99%) and ~2.0 × 10^-4^ M of copper(II) chloride (CuCl_2_, Aldrich, 99%) was mixed with ~1.0 × 10^-3^ M sodium acrylate (H_2_C=CHCO_2_Na, Aldrich, 97%) and stirred at room temperature for 3 days. The resulting deep-purple solution indicated the successful formation of Cu(Au) nanoparticles (NPs). The NP compositions, measured by inductively coupled plasma–optical emission spectroscopy (ICP-OES) and energy-dispersive X-ray spectroscopy (EDS), closely matched the target stoichiometries. Additional details of the synthesis procedure are provided in Ref.(1, 2). Two Cu(Au) nanoalloy compositions, Cu₇₅Au₂₅ and Cu₉₀Au₁₀, were synthesized. Although the present study focuses on Cu₇₅Au₂₅, the Cu₉₀Au₁₀ NPs exhibited similar twin-boundary–mediated chemical separation within their interiors, indicating that this behavior is composition-independent and represents a general phenomenon in Cu(Au) nanoalloys.

**In-situ TEM heating.** In situ vacuum annealing of Cu(Au) NPs was carried out at 450 °C under TEM vacuum. Heating was performed using a Gatan double-tilt heating holder with an FEI Titan 80–300 environmental microscope equipped with a field-emission gun and an image aberration corrector, operated at 300 kV. The temperature was ramped at 100 °C min⁻¹ to the target value, after which the holder was allowed to stabilize for ~10 min to achieve clear atomic-resolution imaging. TEM videos were recorded during the in-situ annealing process.

**Scanning TEM (STEM) characterization.** STEM imaging and EDS line scans were acquired using an FEI Talos F200X microscope equipped with an X-FEG electron source and a Super-X EDS detector, operated at 200 kV.

**Ambient-pressure X-ray Photoelectron Spectroscopy (AP-XPS).** AP-XPS measurements were carried out at the IOS beamline of the National Synchrotron Light Source II (NSLS-II), Brookhaven National Laboratory. The instrument features a reaction chamber with a base pressure below 5 × 10⁻⁹ Torr, a SPECS Phoibos NAP150 hemispherical analyzer, and an integrated sputtering gun for surface preparation. Multiple differential pumping stages between the reaction chamber and the analyzer maintain ultra-high vacuum conditions (< 1 × 10⁻⁷ Torr) within the analyzer while allowing the reaction chamber to operate at pressures of a few Torr. Photoemitted electrons exit the high-pressure chamber through a cone-shaped aperture and pass through the differentially pumped lens system to the energy analyzer, enabling continuous XPS acquisition at pressures up to ~5 Torr. Photon energies of 650 eV (for Au 4f and Cu 3p) and 1150 eV (for Cu LMM) were used in this work. Scofield sensitivity factors were applied to the integrated peak areas within each binding-energy region to determine relative composition changes within the corresponding detection depth.

**Density functional theory (DFT) modeling.** Periodic DFT calculations were performed using the Vienna *Ab initio* Simulation Package (VASP)(3, 4). The PW91 generalized gradient approximation (GGA) (5, 6) was used to describe electron–electron exchange–correlation interactions, and projector augmented-wave (PAW) potentials were employed to treat core-valence interactions(7). A plane-wave cutoff energy of 400 eV was used. Brillouin-zone sampling was performed using a (1×4×4) Monkhorst-Pack k-point mesh(8-10). To validate K-points convergence, segregation energy tests were performed using both (1×4×4) and (1×4×8) meshes, which produced differences of less than 0.01 eV, confirming that the (1×4×4) mesh is sufficient. Structural optimizations were carried out by relaxing all atomic positions until residual forces were below 0.015 eV/Å. Atomic structures were visualized using the Visualization for Electronic and Structure Analysis (VESTA) software package(11).

**Table S1 |** Chemical compositions of the Cu(Au) NPs determined by ICP-OES**.** The measured compositions closely match the target stoichiometries.

|  | Cu_90_Au_10_ | Cu_75_Au_25_ |
| --- | --- | --- |
| Cu (atm.%) | 90.48 | 75.82 |
| Au (atm.%) | 9.52 | 24.18 |

**Table S2 |** Chemical compositions of the Cu(Au) NPs determined by EDS, showing close agreement with the ICO-OES measurements.

|  | Cu_90_Au_10_ NPs | Cu_75_Au_25_ NPs |
| --- | --- | --- |
| Cu (atm.%) | 88.02 | 74.51 |
| Au (atm.%) | 11.98 | 25.49 |

**Figure S1 | EDS analysis of Cu(Au) NPs.** (A) EDS spectrum of Cu_90_Au_10_ NPs, (B) EDS spectrum of Cu_75_Au_25_ NPs. For each spectrum, the electron beam was spread to illuminate hundreds of NPs, ensuring that the measured composition reflects a representative ensemble average.

**Figure S2 | Representative HAADF-STEM images used to assess the prevalence of chemical separation** **in Cu_75_Au_25_ NPs annealed at 450 ºC for ~1h under TEM vacuum.** The occurrence of twinning-facilitated bulk chemical separation was quantified by analyzing ~300 Cu_75_Au_25_ NPs annealed at 400˚C for ~1 h. ~43% of the vacuum-annealed NPs developed distinct Au-rich and Cu-rich lamellae, while the remaining NPs retained a uniform Cu(Au) solid solution with no detectable compositional modulation.

**Figure S3 | Representative HAADF-STEM images of Cu_75_Au_25_ NPs annealed at 450 ºC for ~1h under TEM vacuum, illustrating particles that lack both twinning and compositional modulation.** Analysis of more than 300 vacuum-annealed NPs shows that the presence of a twin boundary is a necessary condition for bulk chemical separation: particles without twin boundaries never exhibit Au-Cu compositional modulation.

**Legends for Supplementary Videos.**

**Supplementary Video 1:** In-situ HRTEM observation of the nucleation of twins in a single-crystal a Cu_75_Au_25_ nanoparticle kept at 450 ˚C. The frame speed is 12× real time.

**Supplementary Video 2:** In-situ HRTEM observation of chemical separation between neighboring twins in a Cu_75_Au_25_ nanoparticle kept at 450 ˚C. The frame speed is 4.5× real time.

**References:**

1.	J. Yin, S. Shan, L. Yang, D. Mott, O. Malis, V. Petkov, F. Cai, M. Shan Ng, J. Luo, B. H. Chen, Gold–copper nanoparticles: nanostructural evolution and bifunctional catalytic sites. *Chem. Mater.* **24**, 4662-4674 (2012).

2.	D. Mott, J. Galkowski, L. Wang, J. Luo, C.-J. Zhong, Synthesis of size-controlled and shaped copper nanoparticles. *Langmuir* **23**, 5740-5745 (2007).

3.	G. Kresse, J. Furthmüller, Efficient iterative schemes for ab initio total-energy calculations using a plane-wave basis set. *Phys. Rev. B* **54**, 11169 (1996).

4.	G. Kresse, J. Furthmüller, Efficiency of ab-initio total energy calculations for metals and semiconductors using a plane-wave basis set. *Comput. Mater. Sci.* **6**, 15-50 (1996).

5.	J. P. Perdew, K. Burke, M. Ernzerhof, Generalized gradient approximation made simple. *Phys. Rev. Lett.* **77**, 3865 (1996).

6.	J. P. Perdew, J. A. Chevary, S. H. Vosko, K. A. Jackson, M. R. Pederson, D. J. Singh, C. Fiolhais, Atoms, molecules, solids, and surfaces: Applications of the generalized gradient approximation for exchange and correlation. *Phys. Rev. B* **46**, 6671 (1992).

7.	G. Kresse, D. Joubert, From ultrasoft pseudopotentials to the projector augmented-wave method. *Phys. Rev. B* **59**, 1758 (1999).

8.	X. Duan, O. Warschkow, A. Soon, B. Delley, C. Stampfl, Density functional study of oxygen on Cu(100) and Cu(110) surfaces. *Phys. Rev. B* **81**, 075430 (2010).

9.	L. Li, G. Zhou, Oxygen subsurface adsorption on the Cu(110)-c(6× 2) surface. *Surf. Sci.* **615**, 57-64 (2013).

10.	Q. Liu, L. Li, N. Cai, W. A. Saidi, G. Zhou, Oxygen chemisorption-induced surface phase transitions on Cu(110). *Surf. Sci.* **627**, 75-84 (2014).

11.	K. Momma, F. Izumi, VESTA 3 for three-dimensional visualization of crystal, volumetric and morphology data. *J. Appl. Crystallogr.* **44**, 1272-1276 (2011).
